# Androgen Receptor Influenced Recurrence Score Correlation in Hormone Positive and HER2 Negative Breast Cancer Indian Patients: A Comparative Approach

**DOI:** 10.14293/genint.15.1.001

**Published:** 2024-07-04

**Authors:** Amit Roy Chowdhury, Somya Saswati Swain, Sandip Kumar Mohanty, Birendranath Banerjee

**Affiliations:** 1 Molecular Stress and Stem Cell Biology Group, School of Biotechnology, KIIT University, Bhubaneswar, Odisha, India; 2 inDNA Centre for Research and Innovation in Molecular Diagnostics, inDNA Life Sciences Private Limited, Bhubaneswar, Odisha, India; 3 Department of Histopathology, inDNA Life Sciences Private Limited, Bhubaneswar, Odisha, India; 4 JBS Haldane Centre of Molecular Medicine, Silicon University, Bhubaneswar, Odisha, India

**Keywords:** Breast cancer, recurrence score, androgen receptor, estrogen receptor, Magee equation, prediction

## Abstract

Breast cancer (BC) recurrence is a major concern for both patients and healthcare providers. Accurately predicting the risk of BC recurrence can help guide treatment decisions and improve patient outcomes for a disease-free survival. There are several approaches and models that have been developed to predict BC recurrence risk. These include derived clinical assays such as genetic profiling (Oncotye Dx, MammaPrint, CanAssist and others), and algorithm derived open access tools such as Magee equations (ME), CTS5 Calculator and Predict Breast cancer. All the clinical assays are well accepted, but affordability and feasibility remain the challenge due to a noteworthy price tag of USD 3000. As per The American Society of Clinical Oncology (ASCO) updates, open access tools are possible substitutes but the availability of limited information on their applicability is a concern. These tools take into consideration the histopathologic parameters and immunohistochemistry (IHC) biomarkers data for estrogen receptor/progesterone (ER/PR), human epidermal growth factor receptor 2 (HER2), and Ki67. The current study focuses on the application of these tools in a subset of 55 Indian BC patients considering the influence of the androgen receptor (AR) IHC expression profile. AR is a potent target and a close interacting neighbor protein to ER and available literature also suggests their crosstalk expression in BC clinical models. Our comparative recurrence scores (RSs) predictive data showed a statistically significant AR expression correlation with average ME scores. No significance was noted across different prediction tools. The findings are suggestive that ME predictive scores are more relevant and informative compared to other online tools and with an additional AR IHC expression analysis the recurrence prediction might prove to be beneficial and feasible to many deprived BC patients.

## Introduction

Breast cancer (BC) is a heterogenous disease with multiple variables including age of diagnosis, hereditary component, co-morbid factors, and histological and immunohistochemical profiles. These factors further classify patients into different molecular sub-types. The low grade luminal-A type predicts a good prognosis with endocrine receptor positivity and HER2/neu negativity with low-to-moderate proliferation index.^[1]^ The distant risk of disease recurrence is a factor often evaluated in an adjuvant clinical management setting for further patient care. Currently, multiple clinically accepted methods are available such as MammaPrint, Oncotype Dx (ODX), Prosigna, MammaTyper and CanAssist. Also, there are open access web-based online tools available as Predict (www.predict.nhs.uk, University of Cambridge, UK) is a prognostication and treatment assessment tool developed using UK cancer registry data. Similarly, CTS5 calculator (www.cts5-calculator.com, Queen Mary University of London, UK) developed from two large UK-based clinical trials (ATAC and BIG1-98) for the prediction of 5–10-year distant recurrence risk in early stage ER positive BC women.^[2,3]^

ODX being the most preferred choice globally and also recognized by the American Society of Clinical Oncology (ASCO) and the National Comprehensive Cancer Network (NCCN), is a commercially developed 21 gene (quantitative real-time polymerase chain reaction) assay with an approximate 3000 USD price tag (Genomic Health, Redwood City, CA, USA).^[4]^ ODX has been demonstrated to be effective and to have predictive use in therapeutic decision-making in patients with estrogen receptor (ER)-positive and lymph node-negative BC. The available literature also supports the use and recurrence prediction of ODX in node-positive BC.^[5]^ The ODX recurrence score (RS) is reported as a number that is divided into either low (<18), intermediate (18–30), or high (>30) recurrence risk categories. With the advent of the prospective clinical trial called Trial Assigning Individualized Options for Treatment (TAILORx), designed to assess the usefulness of oncotype testing has redefined the older algorithm of RS predictions. Subsequently, patients with scores of 0–10 receive only endocrine therapy, patients with oncotype RS 11–25 were randomized to receive either endocrine therapy alone or both endocrine and chemotherapy and patients with scores >25 received both endocrine and chemotherapy. Approximately 15% of ER-positive HER2-negative BC patients are likely to have recurrence at the 5-year timeline in the absence of adjuvant chemotherapy^[6]^ and 85% of patients are likely to be exposed to chemotherapeutic toxicity with minimal clinical benefit. To decrease the demerits of adjuvant chemotherapy, patient stratification based on BC recurrence risk has become an effective aid in individualized BC treatment management.

Practicing advanced molecular and genetic testing for the clinical management of BC or for any other cancer in developing or underdeveloped countries, considering the 3000 USD price tag, is certainly not feasible and is unobtainable for most patients and hence deprives the majority of patients from the benefits of the assay findings. Adjuvant chemotherapy is the standard of care in early BC cases rendering >50% patients at risk from avoidable therapeutic toxicity. In India, a minority (<5%) of BC patients are likely to opt for ODX testing.^[7]^ Current clinical practice in BC employs assessment of tumor size, tumor focality, mitotic rate, tubular differentiation, nuclear morphology, tumor invasion, histologic grade, node involvement followed by hormonal, human epidermal growth factor receptor 2 (HER2), and tumor cell proliferation biomarker testing. Available evidence suggests, standard histopathologic features as described above, are sufficient to yield applicable diagnostic and predictive information of clinical use.^[8–12]^ Taking the routine practice and parameters into consideration, Magee equations (ME) (UPMC, Magee-Womens Hospital, Pittsburg, PA, USA) were developed to estimate the ODX RS in an inexpensive and time efficient manner much suited to the Indian clinical setup. The first ME (ME1), incorporates the Nottingham Score, Ki-67 percentage, tumor size, and H-scores for ER and progesterone receptor (PR), whereas the second ME (ME2) is similar to Equation 1 but excludes Ki-67 and the third ME (ME3) utilizes semi-quantitative immunohistochemical expression levels for ER, PR, HER2, and Ki-67 for assessing the RS precisely.^[13,14]^ ERs play a crucial role in the growth and progression of ER-positive BC as they serve as the target for hormonal therapy, which includes the use of selective ER modulators such as tamoxifen and aromatase inhibitors such as letrozole and exemestane.^[15]^ The androgen receptor (AR) is another hormone receptor (HR) that has gained attention in ER-positive BC management. Studies have shown that the AR is co-expressed with the ER in a subset of ER-positive BCs. This co-expression suggests that targeting the AR may provide an additional therapeutic approach for ER-positive BC. Preclinical studies have demonstrated that inhibiting the AR can suppress the growth of ER-positive BC cells, both *in vitro* and *in vivo*.^[16]^ Furthermore, clinical studies have shown that the presence of AR expression in ER-positive BC is associated with a better prognosis and response to endocrine therapy.

Current literature and evidence portray a mixed opinion on the role of AR in ER+ BCs which has a high reported positivity incidence of 60–80%. In ER+ patients, the AR can either inhibit or promote the BC cell growth, while it predominantly stimulates the cell proliferation in ER− BC patients. The effect can be modulated in an independent manner or in combination with other targeted therapy.^[17]^ Some literature suggests that a tumor with high AR blocks and deregulates ERα expression and promotes metastasis, and endocrine resistance by epigenetically modifying E-cadherin and vimentin expression. Krop *et al.*, in a randomized placebo-controlled phase-II trial showed a sub-set of ER+ patients to benefit from enzalutamide (ENZ), an AR blocker.^[18]^ Whereas, another experimental study showed consensus of a possible link in therapeutic response variation in ER+AR+ BCs with endocrine therapy.^[19]^

In the current study, we have estimated the RS in a heterogenous Indian institutional cohort of 55 HR positive and HER2/neu negative BC patients, irrespective of node status and compared the estimated score with the open source available updated web-based MEs, Predict and CTS5 Calculator to determine the relevance and possible correlation. Also, with the inclusion of an additional immunohistochemical biomarker analysis of AR, we have attempted to visualize any relationship with recurrence risk scale from ME scores, predicting candidate patients into low- and high-risk groups for safely forgoing adjuvant chemotherapy and patients requiring a combination of endocrine and chemotherapy or endocrine therapy alone.

## Material and Methods

A total of 55 BC patients prescribed for recurrence score assessment from January, 2019 to December, 2023 at inDNA Life Sciences were included in this study. Previous clinical summary and an informed consent were obtained individually. Cases were referred from the leading hospitals in Delhi, Uttar Pradesh, Punjab, West Bengal, and Odisha. For each case, hematoxylin and eosin (H&E) stained slides were freshly prepared and reviewed by two board accredited pathologists to validate the histopathological findings, and the Nottingham Score. immunohistochemical staining results for ER (clone EP1; PathnSitu Biotechnologies, Pleasanton, CA, USA), PR (clone EP2; PathnSitu Biotechnologies, Pleasanton, CA, USA), HER2/neu (clone EP3; PathnSitu Biotechnologies, Pleasanton, CA, USA), Ki67 (clone MIB-1; PathnSitu Biotechnologies, Pleasanton, CA, USA) and AR (clone EP120; PathnSitu Biotechnologies, Pleasanton, CA, USA) were revalidated to confirm the initial findings and assessed as per Allred scoring. A semi-quantitative immunohistochemical histologic score (H-score) for ER and PR was calculated as the product of the percentage of positive cells multiplied by the intensity of staining in those cells where the intensity was graded as follows (no staining = 0, weak = 1, moderate = 2, strong = 3) with maximum score being 300. HER2/neu immunostaining was reported according to the 2018 CAP/ASCO guidelines,^[20]^ with 0 and +1 scores considered negative and scores of +3 considered as positive. Other histopathologic parameters such as type of surgery, laterality, tumor size, carcinoma type, histologic grade, and node status were extracted from individual pathology reports of patients.

The updated online Magee equation tool was used to calculate the recurrence scores. The average score of the three equations were recorded to categorize the patients into different risk groups as per criteria described according to the TAILORx trial for assessing ODX scores. Similarly, the available online tools CTS5 Calculator and Predict Beast cancer were also applied to record the scores and risk groups for comparative correlation.

Clinicopathologic features and comparative correlation were calculated using the chi-square test or Fisher’s exact test for categorical or dichotomized variables, or a two sample T-test for continuous variables. DATAtab (web version) and GraphPad Prism (v.8.3.0) statistical calculators were used to record and analyze the data.^[21,22]^ Mean and percentage values were calculated as and where required. Pearson correlation was used to draw significance between recurrence scores. A *P*-value <0.05 was considered statistically significant. Cohen’s d interpretation was used to measure the resultant effect size between two mean differences where a value of 0.2 refers to a small effect, 0.5 refers to a medium effect and a value of >0.8 refers to a large effect. Null hypothesis was rejected in case of *P*-value <0.05 was detected. Structural interaction of estrogen receptor 1 (ESR1) and AR was performed using String network plugin of Cytoscape (v3.10.1) software.^[23]^

## Results

A total of 55 ER/PR positive and HER2/neu negative BC patients irrespective of grade and node status were included in the study for recurrence score prediction. The mean age was 56 years. The majority 38 (69.1%) had undergone modified radical mastectomy (MRM), 4 (7.3%) had breast conservation surgery (BCS), 4 (7.3%) had a lumpectomy, and there was no data for 9 (16.3%) patients. Twenty-eight (50.9%) had a left whereas 20 (36.4%) had a right laterality and, there was no data for 7 (12.7%) patients. Recorded histopathologic features represented a mean tumor size of 3.07 cm (T1 – 27; 49.1%, T2 – 22; 40%, and T3 – 6; 10.9%). Considering the pT3 subset of ER+ BC patients (n = 06), only 1 (3.1%) patient presented with AR+ expression, whereas the majority of patients belonged to the pT1 (n = 17; 51.5%) and pT2 (n = 15; 45.4%) subgroups. Thirteen (23.6%) patients presented Nottingham histologic grade-I, 34 (61.8%) patients presented grade-II and 8 (14.6%) patients presented grade-III. Histologic findings revealed patients with invasive ductal carcinoma, no special type (85.5%), invasive lobular carcinoma (1.8%), mixed carcinoma (3.6%), metaplastic carcinoma (1.8%), and mucinous carcinoma (hypercellular variant), invasive ductal carcinoma (micropapillary, acinar variant), were classified as others (7.3%). Twenty-four (43.6%) patients had low proliferative index (Ki67 IHC: <10%) and 31 (56.4%) patients had comparatively high proliferative index (Ki67 IHC: >10%). Similarly, 16 patients presented node metastasis of more than or equal to 1 lymph node (29.1%), out of which 9 (27.3%) were associated with AR+ expression. Data on lymph node status was not available for 14 (25.4%) patients. Overall, no statistical significance was noted with histologic parameters and AR+ expression.

IHC study revealed 33 (60%) cases to be AR positive (Table 1).

**Table 1:** Comparative clinico-pathologic characteristics of BC cases included in the study with AR positive cases.

Parameter	Overall patients (n = 55)	AR+ (n = 33)	*P*-value
Age (years)			
<40	05 (9.1%)	01 (3.1%)	0.177
40–60	30 (54.5%)	18 (54.5%)	
>60	20 (36.4%)	14 (42.4%)	
Tumor size (cm)			
<2.5	27 (49.1%)	17 (51.5%)	0.084
2.5–5.0	22 (40.0%)	15 (45.4%)	
>5.0	06 (10.9%)	01 (3.1%)	
Nottingham grade			
Grade I	13 (23.6%)	10 (30.3%)	0.523
Grade II	34 (61.8%)	21 (63.6%)	
Grade III	08 (14.6%)	02 (6.1%)	
Ki67 index			
<10%	24 (43.6%)	11 (33.3%)	0.146
>10%	31 (56.4%)	22 (66.7%)	
Node status			
Negative	25 (45.5%)	13 (39.4%)	0.417
Positive	16 (29.1%)	09 (27.3%)	
No record	14 (25.4%)	11 (33.3%)	
Histologic type			
Invasive ductal carcinoma (NST)	47 (85.5%)	30 (90.8)	0.161
Invasive lobular carcinoma	01 (1.8%)	01 (3.1%)	
Mixed carcinoma	02 (3.6%)	0 (–)	
Metaplastic carcinoma	01 (1.8%)	0 (–)	
Others	04 (7.3%)	02 (6.1%)	
Surgical procedure			
Modified radical mastectomy (MRM)	38 (69.1%)	23 (69.7%)	0.317
Breast conservation surgery (BCS)	04 (7.3%)	04 (12.1%)	
Lumpectomy	04 (7.3%)	03 (9.1%)	
No record	09 (16.3%)	03 (9.1%)	
Laterality			
Left	28 (50.9%)	18 (54.6%)	0.750
Right	20 (36.4%)	12 (36.3%)	
No record	07 (12.7%)	03 (9.1%)	

**Figure 1:**
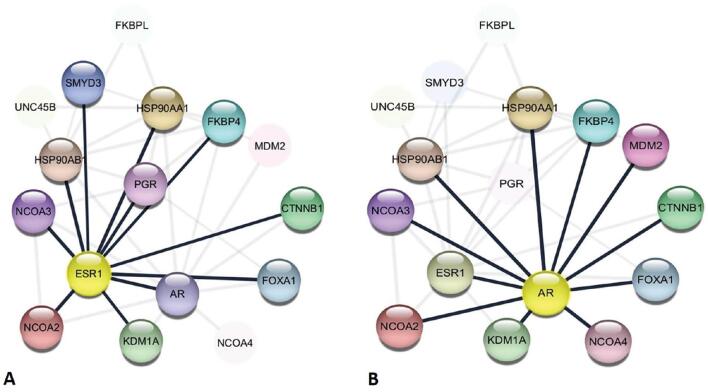
Structural string network analysis of associated (A) ESR1 and (B) AR protein interaction highlighting first neighbors.

A close association (score: 0.700) string network analysis for ESR1 (Figure 1A) and AR (Figure 1B) proteins revealed both to be ligand dependent nuclear proteins closely interacting strong first neighbors.

**Figure 2:**
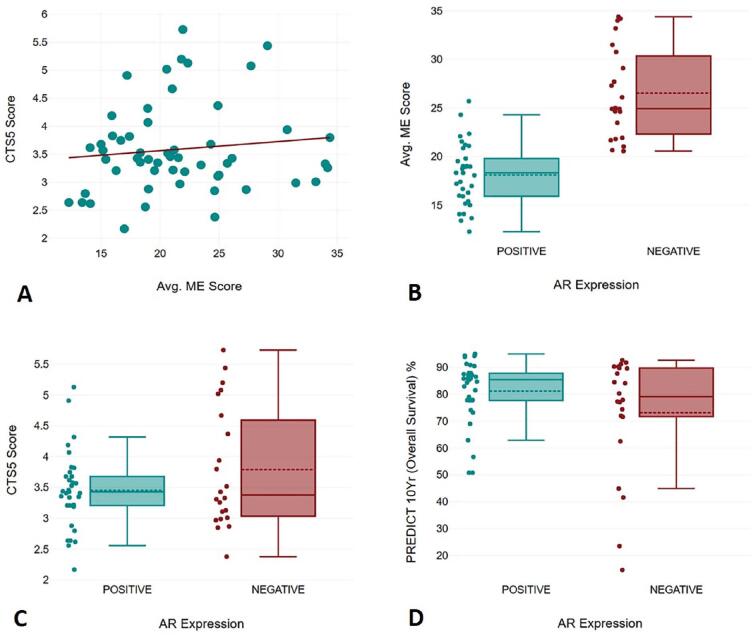
(A) Scatter plot representative of low positive correlation between Avg. ME score and CTS5 score; (B) box plot representative of significance between Avg. ME score and AR expression; (C) box plot representative of significance between CTS5 score and AR expression; (D) box plot representative of significance between PREDICT Breast cancer (10-year survival %) and AR expression.

Furthermore, individual patient data was plotted via open web-based tools to generate average ME scores, CTS5 scores, Predict Breast cancer 10-year survival rate and correlation was drawn with AR expression. The result of the Pearson correlation showed that there was a low, positive correlation between average (Avg.) ME Score and CTS5 Score (Figure 2A). The correlation between Avg. ME Score and CTS5 Score was not statistically significant, r = 0.12, *P* = 0.402. A two-tailed t-test for independent samples showed that the difference between AR+/− expression with respect to the dependent variable Avg. ME Score was statistically significant, (t = −7.34, *P* = <0.001), 95% confidence interval (CI) (−10.75, −6.09). Thus, the null hypothesis between the two groups was rejected (Figure 2B). The Cohen’s d value of 2.02 represents a large effect size between the groups. A two-tailed t-test for independent samples (equal variances not assumed) showed that the difference between positive and negative AR expression with respect to the dependent variable CTS5 Score was not statistically significant, t (32.44) = −1.42, *p* = 0.164, 95% CI [−0.82, 0.14]. Thus, the null hypothesis in the mean value between the two groups was accepted (Figure 2C). The Cohen’s d value of 0.39 represents a small effect size. A two-tailed t-test for independent samples (equal variances not assumed) showed that the difference between positive and negative AR expression with respect to the dependent variable Predict Breast cancer 10 year (overall survival %) was not statistically significant, t (28.75) = 1.54, *P* = 0.135, 95% CI [−2.65, 18.63]. Thus, the null hypothesis between the two groups was accepted (Figure 2D). The Cohen’s d value of 0.42 represents a small effect size.

A significant influence of AR expression was observed on average ME scores with AR+ patients falling into the considerably low ME score group while AR− patients predominantly belonged to the high ME score group. No significant difference was noted with respect to the age of the patients in both the groups. It was noted that the chi-square test presented a statistically significant difference (*P* = <0.0001) among categorical predicted risk groups between average ME and CTS5 predicted recurrence scores (Table 2).

**Table 2:** Distribution and correlation between individual recurrence score groups based on Avg. ME and CTS5 calculator scores.

Recurrence score (RS) prediction tool	Low risk	Intermediate risk	High risk	*P*-value
Avg. ME rec. score	16.4% (n = 09)	83.6% (n = 46)	–	<0.0001
CTS5 calculator score	27.3% (n = 15)	49.1% (n = 27)	23.6% (n = 13)	

## Discussion

Integrating clinicopathological parameters with molecular information is a standard practice in BC recurrence risk prediction. Several online tools are available to estimate the same. The current study employed three different online accessible tools to estimate and compare significance of the predicted scores in a set of 55 eligible patients. Available evidence has shown that AR is a tumor suppressor and that its expression is associated with improved prognosis in ER+ BC. Although, the role of AR in ER/PR positive BC has been controversial, growing and advancing literature shows a possible functional AR-ER crosstalk in ER positive BC. In the context of the AR/ER crosstalk, several studies have focused on the role of ENZ, an AR inhibitor (commonly used for prostate cancer) in BC models. In cases of low AR expression, ENZ directly functioned as an ERα antagonist via cell growth inhibition by ENZ in BC with low AR expression was independent of AR and instead dependent on ER. Whereas, in AR-high BC models, AR repressed ERα signaling and ENZ promoted ERα signaling by antagonizing AR.^[19]^

The actions of these steroids are achieved through gene regulation via nuclear steroid receptors acting as transcriptional factors by binding to enhancer regions, including the estrogen-responsive element (ERE) and androgen-responsive element (ARE). String network analysis also suggests AR-ER as close interacting proteins. ER and AR are co-expressed and directly interact with each other in BC cells. Also, pre-clinical evidence shows that anti-androgens that inhibit AR nuclear localization affect both AR and ER and could be effective in combination with current BC therapy options. AR is an emerging feasible biomarker in BC management.^[24,25]^

Integrating AR expression in the present study revealed a statistically significant difference with ME predicted scores with AR+ cases (n = 33; 60%) correlated with a comparatively low RSs with respect to AR− cases (n = 22; 40%). No correlation was drawn with CTS5 calculated and Predict Breast cancer scores with AR expression. It is important to note that CTS5 calculator only takes tumor size, grade, age, and node status into consideration while Predict Breast cancer considers age, DCIS/LCIS status, menopausal status, ER, HER2, Ki67 status, tumor size, grade, and node status for prediction of 10-year disease free survival rate. However, updated ME’s alone employs the Nottingham Score, ER/PR calculated histologic (H) score, HER2 status, tumor size, and Ki67 data. The chi-square test between Avg. ME scores and CTS5 calculated scores predicted a strong statistically significant difference (*P* = <0.0001) between individual low, intermediate, and high-risk groups indicating the values to be independent.

As per the literature, the current findings suggest it to be the only study examining the association of AR IHC expression with RS predictions via three online tools. The ME RS prediction algorithm is the only online RS prediction tool with an elaborative application of clinicopathologic parameters to assess a reliable recurrence score comparable to ODX prediction scores.^[26]^ Similarly, preclinical data also suggests that the expression of AR may modify clinical outcomes in early BC with improved prognosis in ER+ and poorer prognosis in ER− disease.^[27,28]^ The findings are preliminary but suggest a positive correlation between ME scores and AR+ expression. Hence, AR poses the impedance of elevating the RS prediction models in a more precise manner. This not only provides varied BC management options with AR inhibitors but may also be predictive in RS estimation. To conclude, the current findings suggest that ME predictive scores are more relevant and informative compared to other online tools and with an additional AR IHC expression analysis the recurrence prediction might prove to be beneficial and feasible to many deprived BC patients. A 5- and 10-year follow-up with a patient cohort for recurrence free survival will consolidate the findings and efficacy of the various open access tools assessed.

## Data Availability

All the relevant patient information and data will be made available upon request by the corresponding author.
